# Efficacy and Safety of Quadruplet Therapy in Newly Diagnosed Transplant‐Eligible Multiple Myeloma: A Systematic Review and Meta‐Analysis

**DOI:** 10.1002/cnr2.70171

**Published:** 2025-04-02

**Authors:** Aneeta Channar, Syed Arsalan Ahmed Naqvi, Muhammad Ali Khan, Arifa Bibi, Akshat Saxena, Nikita Tripathi, Ahmad Iftikhar, Ammad Raina, Kaneez Zahra Rubab Khakwani, Irbaz Bin Riaz, Muhammad Husnain

**Affiliations:** ^1^ Division of Hematology and Medical Oncology Mayo Clinic Phoenix Arizona USA; ^2^ The University of Arizona Tucson Arizona USA; ^3^ Canyon Vista Medical Center Sierra Vista Arizona USA

**Keywords:** anti‐CD38 antibody, daratumumab, isatuximab, multiple myeloma

## Abstract

**Background:**

The treatment landscape for multiple myeloma continues to evolve. Recently, the addition of anti‐CD38 monoclonal antibodies (mAbs) to the triplet regimen, comprising a proteasome inhibitor, an immunomodulatory agent, and a steroid, for transplant‐eligible newly diagnosed multiple myeloma (TENDMM) has shown promising results.

**Aims:**

To evaluate the overall efficacy and safety of quadruplet therapy with an anti‐CD38 mAb compared to a triplet regimen.

**Methods:**

A systematic search of Medline, Scopus, and EMBASE databases from inception to July 2024 identified relevant randomized controlled trials (RCTs). Efficacy and safety outcomes were derived using random‐effects meta‐analysis. Summarized outcomes include hazard ratios (HR) for progression‐free survival (PFS) and overall survival (OS), odds ratios (OR) for response rates, measurable residual disease (MRD) negativity rate, and grade 3 or higher adverse events (G ≥ 3 AEs).

**Results:**

Five RCTs involving 2963 patients were included. A statistically significant PFS was observed for quadruplet therapy when compared to the triplet regimen (HR 0.44; 95% Confidence Interval [CI] 0.35–0.56). PFS benefit was consistent for the standard risk (SR) group (HR 0.38; 95% CI 0.27–0.52) and high risk (HiR) group (HR 0.62; 95% CI 0.41–0.92). No statistically significant benefit was observed for OS (HR 0.55; 95% CI 0.28–1.08). A statistically significant benefit was observed for the overall response rate (OR 1.77; 95% CI 1.02–3.06) and MRD negativity rate (OR 2.67; 95% CI 1.79–3.99). No significant differences were observed for G ≥ 3 AE (OR 1.21; 95% CI 0.92–1.58), lymphopenia (OR 1.09; 95% CI 0.62–1.89), and anemia (OR 1.06; 95% CI 0.83–1.37). However, a significantly increased risk was observed for all‐grade thrombocytopenia (OR 1.64; 95% CI 1.37–1.97), neutropenia (OR 2.24; 95% CI 1.67–3.02) and infections (OR 1.88; 95% CI 1.07–3.31).

**Conclusion:**

Quadruplet therapy demonstrated a favorable efficacy and safety profile, with consistent benefit across subgroups. The findings support its potential as the new standard of care for TENDMM.

## 1 Introduction

Multiple myeloma (MM) is the second most common hematological cancer [1], with approximately 32 000 new cases diagnosed each year in the United States [2]. Although the survival for MM patients has improved significantly over the past decade, reaching a median of 8–10 years [3], the disease remains incurable, underscoring the need for novel therapeutic approaches to further enhance patient outcomes [4]. Risk stratification and eligibility for autologous stem cell transplantation (ASCT) pose important caveats to the treatment of newly diagnosed multiple myeloma [5]. Historically, the triplet therapy consisting of a proteasome inhibitor, an immunomodulatory agent, and a steroid had long been the standard of care for transplant‐eligible newly diagnosed multiple myeloma (TENDMM) [6]. However, in recent years, the therapeutic landscape of MM has evolved with the introduction of multiple monoclonal antibodies (mAbs). Notably, anti‐CD38 mAbs, Daratumumab (Dara) and Isatuximab (Isa) have demonstrated substantial efficacy, which has led to their initial FDA approval for relapsed/refractory multiple myeloma (RRMM) [7].

CASSIOPEIA [8] was the first phase three randomized controlled trial (RCT) to provide evidence for the efficacy of Dara‐based induction and consolidation in TENDMM. Subsequently, trials such as GRIFFIN [9] and PERSEUS [10] have provided further evidence that Dara‐based regimen yields prolonged and deeper responses in TENDMM. The earliest evidence for the incorporation of Isa comes from the GMMG‐HD 7 trial [11], followed by the Iskia trial [12], both of which reported that the Isa‐based arms yielded superior responses. Sustained deep responses to initial therapy are in turn associated with improved survival outcomes, particularly an increase in progression‐free survival (PFS) [13]. Previous meta‐analyses, conducted since 2019, have evaluated Dara‐based quadruplets in frontline MM [14, 15, 16, 17]. However, with rapidly evolving evidence, this systematic review and meta‐analysis aims to provide a comprehensive summary of the overall efficacy and safety of quadruplet therapy with anti‐CD38 (Dara or Isa) in TENDMM, incorporating the latest reports from clinical trials. Our analysis focuses on the transplant‐eligible cohort to guide clinicians in selecting the best possible regimen for this group. Additionally, our study provides an in‐depth evaluation of efficacy across clinically relevant subgroups, offering valuable insights into optimizing treatment strategies for TENDMM patients.

## 2 Methods

This systematic review is reported in accordance with the Preferred Reporting Items for Systematic Reviews and Meta‐analysis (PRISMA) guidelines [18]. A protocol was designed before the research began; however, the review protocol has not been registered.

### 2.1 Literature Search and Study Selection

The search strategy was designed with input from study investigators. Ovid Medline, EMBASE, and SCOPUS were searched from each database's inception (MEDLINE, 1946; EMBASE, 1974; SCOPUS, 2004) through July 1, 2024, to retrieve all phase 2 or phase 3 RCTs comparing quadruplet therapy with anti‐CD38 monoclonal antibody (Dara or Isa) to the triplet regimen in TENDMM. Studies involving patient populations that had received prior interventions were excluded. In addition, major oncology proceedings (American Society of Clinical Oncology, American Society of Hematology, and European Society for Medical Oncology) were manually searched from 2016 to 2024 for published abstracts. The inclusion criteria were limited to English‐language articles only. Following de‐duplication, two reviewers (A.C and S.A.A.N) performed an independent screening of titles and abstracts identified by the search, followed by a detailed full‐text evaluation for eligibility. Any disagreement between the two reviewers was resolved by input from a third reviewer (M.H). The detailed search strategy is presented in eMethods in the supplement.

### 2.2 Data Extraction

Two independent reviewers (A.C and M.H) extracted the data using standardized data collection forms. Discrepancies were resolved by a third reviewer (I.B.R). Retrieved data included trial characteristics such as publication year, authors, sample size, interventions, duration of follow‐up, and reported outcomes. Additionally, baseline characteristics of the patient population (age, sex, race/ethnicity, and cytogenetic risk category) were also extracted.

### 2.3 Outcomes of Interest

The efficacy outcomes included PFS, overall survival (OS), measurable residual disease (MRD) negativity rate, overall response rate (ORR), rate of stringent complete response (sCR), very good partial response or better (VGPR or better), and complete response or better (CR or better). Safety outcomes included the risk of grade 3 or higher adverse events (G ≥ 3), cytopenia, and infections. We extracted the MRD negativity rate with a threshold of 10^−5^, as it was consistently reported in the trials. Outcomes were defined in accordance with the definitions provided in the included trials (Table S2 in supplement). If multiple publications were available for a trial, data from the publication with the longest follow‐up were used to derive efficacy outcomes.

### 2.4 Risk of Bias

Risk of bias was assessed for each outcome using the Cochrane Collaboration's tool [19]. Two reviewers independently evaluated trial quality (A.C and S.A.A.N), with disagreements resolved by referring to a third reviewer (M.H).

### 2.5 Statistical Analysis

A DerSimonian‐Laird random‐effects meta‐analysis was conducted to estimate the summary effect size. For survival outcomes, including PFS and OS, an inverse variance approach was used to pool pre‐computed hazard ratios (HR) with 95% confidence intervals (CI) after logarithmic transformation. For raw binary outcomes, which included both efficacy and safety outcomes, the Mantel–Haenszel approach was used to pool and compute odds ratios with 95% CIs. The Cochrane *Q* test was used to assess statistically significant heterogeneity not explained by chance, and the *I* ^2^ statistical test was used to quantify total observed variance due to between‐study variances. An *I* ^2^ statistic greater than 50% indicates substantial heterogeneity. A 2‐sided *p*‐value was used, with results considered significant if *p* < 0.05. Pre‐specified subgroup analyzes for PFS were performed by cytogenetic risk group. Additional subgroup analyzes were performed for MRD negativity by cytogenetic risk, number of abnormal cytogenetic features (Double‐hit vs. Single‐hit), International Staging System (ISS) score, Eastern Cooperative Oncology Group (ECOG) score, and type of MM (IgG vs. non‐IgG). A *p*‐value of interaction (*P*_int_) was computed to detect significant interaction between the subgroups, with a value of < 0.10 considered statistically significant. The statistical analyzes were conducted in R (version 4.4.1, R Project for Statistical Computing).

### 2.6 Certainty of Evidence

GRADE approach (Grading of Recommendations, Assessment, Development, and Evaluation) was used to evaluate the certainty of evidence. Two reviewers (A.C and S.A.A.N) independently ascertained the certainty in estimates, with disagreement resolved by a third reviewer (M.H).

## 3 Results

A total of 11 762 studies were identified through the search strategy for the databases. An additional conference abstract was identified through a manual search [12]. After thorough screening, five trials (seven references) [8, 9, 10, 11, 12, 20, 21] with a total of 2963 patients were included in the systematic review and meta‐analysis. The process of study selection is outlined in Figure S1 in the supplement.

### 3.1 Baseline Study and Population Characteristics

Among the five trials included, three evaluated daratumumab‐based regimens: two trials investigated Dara in combination with bortezomib, lenalidomide, and dexamethasone (RVd), while one examined daratumumab with bortezomib, thalidomide, and dexamethasone (VTd). The remaining two trials assessed Isa‐based regimens: one in combination with carfilzomib, lenalidomide, and dexamethasone (KRd), and the other with RVd. Baseline patient characteristics were comparable across studies. From the reported data in the trials, the patient population consisted of 1579 males (56.39%) and 1221 females (43.61%). The trial participants included 1468 individuals reported as White (96.3%), 39 as Black (2.6%), and 13 as Asian (0.9%). High‐risk cytogenetics (HiR) were reported in 373 patients (17.2%), while 1795 patients (82.7%) were categorized as standard risk (SR). The median age of the patient population was 59.5 years (Interquartile range [IQR] 59–60.25), and the median follow‐up time was 21 months (IQR 18.8–47.5). Additional baseline characteristics are shown in Table 1.

**TABLE 1:** Characteristics of the included trials.

Trial	Phase	Median follow‐up (months)	Arm	No of patients	Median Age (Range)	Race: *n* (%)	Male: *n* (%)	Female: *n* (%)	High‐risk: *n* (%)	Standard‐risk: *n* (%)
Moreau 2019, (CASSIOPEIA) [8].	3	18.8	D‐VTd	543	59 (22–65)	NA	316 (58.2)	227 (41.8)	82 (15)	460 (85)
			VTd	542	58 (26–65)		319 (58.9)	223 (41.1)	86 (16)	454 (84)
Goldschmidt, 2022 (GMMG‐HD 7) [11].	3	4.1	Isa‐RVd	331	59 (54–64)	White 327 (99)	204 (62)	127 (38)	58 (18)	254 (77)
						African 1 (< 1)				
						Arabic 1 (< 1)				
						Asian 2 (< 1)				
			RVd	329	60 (54–65)	White 327 (99)	206 (63)	123 (37)	66 (20)	234 (71)
						African 1 (< 1)				
						Arabic 0				
						Asian 1 (< 1)				
Voorhees, 2023 (GRIFFIN) [9, 20].	2	49.6	D‐RVd	104	59 (51–65.5)	White 85 (82)	58 (56)	46 (44)	16 (16)	82 (84)
						Black 14 (13)				
						Asian 0				
						Other 2 (2)				
			RVd	103	61 (54–66)	White 76 (74)	60 (58)	43 (42)	14 (14)	83 (86)
						Black 14 (13)				
						Asian 0				
						other 2 (2)				
Gay, 2023 (IsKia Trial) [12].	3	21	IsaKRd	151	61 (55–66)	NA	NA	72 (48)	25 (18)	115 (82)
			KRd	151	60 (54–63)		NA	67 (44)	26 (19)	113 (81)
Sonneveld, 2024 (PERSEUS) [10].	3	47.5	D‐VRd	355	61 (32–70)	White 330 (93)	211 (59.4)	NA	76 (21.4)	264 (74.4)
						Black 5 (1.4)				
						Asian 4 (1.1)				
						Other 4 (1.1)				
			VRd	354	59 (31–70)	White 323 (91.2)	205 (57.9)	NA	78 (22)	266 (75.1)
						Black 4 (1.1)				
						Asian 6 (1.7)				
						Other 3 (0.8)				

Abbreviations: D, daratumumab; d, dexamethasone; Isa, isatuximab; K, carfilzomib; R, lenalidomide; T, thalidomide; V, bortezomib.

### 3.2 Efficacy Outcomes

#### 3.2.1 Progression‐Free Survival

A total of three trials reported PFS [8, 10, 20]. Quadruplet therapy was associated with a statistically significant improvement in PFS (HR 0.44; 95% CI 0.35–0.56; *I* ^2^: 0%) when compared to triplet therapy. The results of PFS analyses are shown in Figure S3 in the supplement.

On subgroup analysis by cytogenetic risk, quadruplet therapy was associated with a statistically significant improvement in PFS for both HiR (HR 0.62; 95% CI 0.41–0.92) and SR group (HR 0.38; 95% CI 0.27–0.52). There was a statistically significant difference in treatment effect by cytogenetic risk (*P*_int_: 0.0). Additional details can be found in Figure S1 in the supplement.

#### 3.2.2 Overall Survival

A total of two trials reported OS [8, 20]. No statistically significant difference in OS was observed between quadruplet therapy and triplet therapy (HR 0.55; 95% CI 0.28–1.08; *I* ^2^: 28%). OS results are available in Figure S4 in the supplement.

#### 3.2.3 Response Rates

Quadruplet therapy was associated with a statistically significant improvement in ORR (OR 1.77; 95% CI 1.02–3.06; *I* ^2^: 35%), sCR (OR 1.72; 95% CI 1.07–2.77; *I* ^2^: 84%), CR or better (OR 2.04; 95% CI 1.28–3.25; *I* ^2^: 79%) and VGPR or better (OR 1.97; 95% CI 1.31–2.96; *I* ^2^: 66%). Quadruplet therapy was associated with a statistically significant improvement in MRD negativity rate (OR 2.67; 95% CI 1.79–3.99; *I* ^2^: 83%). The detailed results of response rates are presented in Figures S5–S9 in the supplement. Quadruplet therapy was associated with a statistically significant improvement in MRD negativity rate for both HiR (OR 2.01; 95% CI 1.41–2.86) and SR group (OR 2.60; 95% CI 1.84–3.69). There was no statistically significant difference in treatment effect by cytogenetic risk group (*P*_int_: 0.31). Quadruplet therapy was associated with a statistically significant improvement in MRD negativity rate in the double‐hit (OR 4.21; 95% CI 1.06–16.70) and single‐hit HiR group (OR 1.86; 95% CI 0.95–3.64). There was no statistically significant difference in treatment effect between the two groups (*P*_int_: 0.29). Additional subgroup analyzes have been presented for MRD negativity rate in Figures S17–S24 in the supplement.

### 3.3 Safety Outcomes

No statistically significant difference was observed for G ≥ 3 AEs between quadruplet and triplet therapy (OR 1.21; 95% CI 0.92–1.58; *I* ^2^: 49%). However, quadruplet therapy was associated with a statistically significant increase in the risk of all‐grade thrombocytopenia (OR 1.64; 95% CI 1.37–1.97; *I* ^2^: 0%), neutropenia (OR 2.24; 95% CI 1.67–3.02; *I* ^2^: 62%) and infections (OR 1.88; 95% CI 1.07–3.31; *I* ^2^: 82%). There was no statistically significant difference between quadruplet and triplet therapy with regard to all‐grade lymphopenia (OR 1.09; 95% CI 0.62–1.89; *I* ^2^: 79%), and anemia (OR 1.06; 95% CI 0.83–1.37; *I* ^2^: 0%). Safety analyses are presented in Figures S10–S15 in the supplement.

### 3.4 Risk of Bias

The overall risk of bias was judged as “low” or “some concerns”. For the GRIFFIN trial [9], some concerns were noted due to reported protocol deviations. For the Iskia trial [12], some concerns were noted in three domains as the risk of bias could not be ascertained from the limited data in the published abstract. The domain‐wise assessment is presented in Figure S2 in the supplement.

### 3.5 Certainty of Evidence

The certainty of evidence ranged from high to moderate, except for OS, which was rated low primarily due to serious imprecision and inconsistency, and for G ≥ AEs, which was rated low due to serious imprecision. A detailed assessment is presented in Table 2.

**TABLE 2:** Certainty of evidence according to the GRADE approach.

Certainty assessment					No of patients		Effect		Certainty
№ of studies	Risk of bias	Inconsistency	Indirectness	Imprecision	Quadruplet therapy	Triplet therapy	Relative (95% CI)	Absolute (95% CI)	
Progression free survival									
3	Not serious	Not serious	Not serious	Not serious	1002	999	**HR 0.63** (0.42 to 0.96)	**294 more per 1000** (from 209 more to 368 more)	⨁⨁⨁⨁ High
Overall survival									
2	Not serious	Not serious	Not serious	Serious^b^	647	645	**HR 0.55** (0.28 to 1.008)	**35 fewer per 1000** (from 57 fewer to 6 more)	⨁⨁◯◯ Low
Overall response rate									
3	Not serious	Not serious	Not serious	Serious^b^	945/998 (94.7%)	909/994 (91.4%)	**OR 1.49** (1.05 to 2.10)	**26 more per 1000** (from 4 more to 43 more)	⨁⨁⨁◯ Moderate
MRD negativity rate									
5	Not serious	Serious^a^	Not serious	Not serious	914/1484 (61.6%)	578/1479 (39.19%)	**OR 2.67** (1.79 to 3.99)	**241 more per 1000** (from 144 more to 328 more)	⨁⨁⨁◯ Moderate
Grade 3 or higher adverse events									
4	Not serious	Not serious	Not serious	Serious^b^	865/1316 (65.7%)	832/1315 (63.3%)	**OR 1.21** (0.92 to 1.58)	**43 more per 1000** (from 20 fewer to 99 more)	⨁⨁◯◯ Low

Abbreviations: CI, confidence interval; HR, hazard ratio; OR, odds ratio.

^a^ Statistically significant heterogeneity with non‐overlapping confidence intervals.

^b^ Serious imprecision due to wide confidence intervals.

## 4 Discussion

Frontline treatment for MM has progressed from doublet regimens to triplets, and now the incorporation of quadruplets represents the latest advancement. Our study uniquely synthesizes evidence from five trials assessing anti‐CD38 mAbs (Dara or Isa) in a comprehensive cohort of 2963 patients, addressing critical clinical questions regarding optimal therapy in TENDMM. The findings suggest that the addition of anti‐CD38 mAbs is associated with superior PFS compared to the standard‐of‐care triplet regimen.

The available evidence indicates that the cytogenetic risk group may be a key factor in frontline treatment. Quadruplet therapy demonstrated efficacy in prolonging PFS across both standard‐risk and high‐risk cytogenetic groups. Our results did not show an overall OS advantage. Notably, OS data remain immature for many of the included trials [10, 11, 12] with longer‐term follow‐up ongoing. In terms of response rates, our analysis revealed significantly improved rates of ORR, sCR, CR or better, and VGPR or better. Furthermore, the odds of achieving MRD‐negative status were significantly higher with quadruplet therapy, and this benefit was consistent regardless of cytogenetic risk. The toxicity profile of quadruplet therapy was acceptable, with no significant difference in the risk of grade ≥ 3 adverse events (AEs) compared to the triplet regimens. However, we noted an increased risk of all‐grade infections, neutropenia, and thrombocytopenia. Given the generally healthier status of transplant‐eligible patients, this toxicity profile appears manageable.

Despite advances in novel therapies, outcomes for the HiR population remain suboptimal due to an increased risk of early recurrence [22]. Clinical trials specifically targeting responses in HiR MM are lacking, and large phase 3 studies have traditionally enrolled a small subset of HiR patients, affecting statistical significance. Furthermore, prognosis for HiR patients differs based on the number of cytogenetic abnormalities [21]. Hence, there is a need for reliable surrogate endpoints to accelerate the adoption of newer approaches for this vulnerable subgroup. MRD negativity rate is one such endpoint that has established its prognostic value in the setting of MM. A meta‐analysis by Landgren et al. [23], which combined eight studies with 4907 newly diagnosed patients, demonstrated a significant relationship between MRD negativity and PFS (OR 4.02; 95% CI 2.57–5.46). Given the limited PFS data in our analysis, the pooled effect of MRD negativity warrants careful consideration. Our subgroup analysis revealed significant benefits for MRD negativity in both double‐hit and single‐hit patients; however, these findings should be interpreted with caution due to the small sample size as only two of the five included trials reported data for this important subgroup. Further subgroup analysis based on MRD negativity showed consistent benefit for quadruplet therapy across gender, ISS stages, type of MM, as well as ECOG performance status, with no subgroup differences observed. In a real‐world study by Joseph et al. [24]. Dara‐based quadruplet therapy in comparison with triplet therapy (D‐RVd vs. RVd) revealed an improved 2‐year PFS in both HiR (83% vs. 69%) and SR groups (94% vs. 84%). Even though the follow‐up period was shorter for the D‐RVd cohort (19.1 vs. 90 months), the results demonstrated an early benefit, supporting our findings that an upfront intensive treatment approach with an anti‐CD38 mAb‐based quadruplet regimen may achieve prolonged remission in both HiR and SR groups. Based on the existing evidence of an improved MRD and PFS, we suggest that the incorporation of anti‐CD38 mAb‐based quadruplets may provide the best outcome in the HiR subgroup.

Current National Comprehensive Cancer Network (NCCN) guidelines recommend Dara‐based quadruplet as the preferred regimen in TENDMM, with an Isa‐based regimen also categorized as a recommended option [25].

## 5 Strengths and Limitations

This meta‐analysis offers significant insights to inform clinical practice, underpinned by rigorous methodology and quality assessment, integrating the most current evidence from recent trials. However, our analysis is limited by a small number of trials and the use of only study‐level data. There was a limited follow‐up, with variable follow‐up durations between the trials. The included trials constituted varied backbone regimens, which differed with regard to the duration of induction and consolidation therapy. Additionally, maintenance regimens also varied between the trials, which may have confounded the ability to determine the independent contribution of the regimen during each phase of the treatment.

Only two trials reported OS, with future studies required to validate long‐term follow‐up results. There was no homogeneous definition of HiR MM across the trials (Table S3 in supplement). Additionally, there was inconsistent reporting of outcomes across the different trials and limited data for the cytogenetic subgroups (Table S1 in supplement). Due to the ongoing nature of the trials, data was insufficient to pool outcomes following maintenance therapy. Finally, the small number of included studies precluded formal assessment of publication bias.

## 6 Conclusions

The results support quadruplet therapy with anti‐CD38 mAbs as the new standard of care for TENDMM. This approach appears particularly promising for HiR patients, offering improved MRD and PFS outcomes. Anti‐CD38 mAb‐based regimens are effective and well tolerated in TENDMM. However, continued efforts are required to optimize disease control in HiR subgroups, particularly among double‐hit patients.

## Author Contributions

**Aneeta Channar:** data curation, methodology and manuscript writing. **Aneeta Channar, Syed Arsalan Ahmed Naqvi, Irbaz Bin Riaz, Muhammad Husnain:** methodology, statistical analysis, and manuscript review/editing. **Aneeta Channar, Arifa Bibi, Akshat Saxena, Nikita Tripathi:** conceptualization, initial screening, and data curation. **Muhammad Ali Khan**, **Arifa Bibi, Ammad Raina, Kaneez Zahra Rubab Khakwani:** initial screening and data curation. **Muhammad Husnain:** conceptualization and manuscript review/editing. **Ahmad Iftikhar, Irbaz Bin Riaz:** manuscript review/editing. **Irbaz Bin Riaz, Ahmad Iftikhar, Muhammad Husnain:** conceptualization, data curation, methodology, supervision, and manuscript review/editing. All authors reviewed and edited the final version of the manuscript.

## Disclosure

The authors have nothing to report.

## Conflicts of Interest

The authors declare no conflicts of interest.

## Supporting information


**Data S1.** Supporting Information.

**Data S2.** Supporting Information.

## Data Availability

The datasets extracted and analyzed during the current study are available from the corresponding author upon request.
