# Supplementary material for: Efficacy and Safety of Quadruplet Therapy in Newly Diagnosed Transplant‐Eligible Multiple Myeloma: A Systematic Review and Meta‐Analysis
Source: Cancer Rep (Hoboken). 2025 Apr 2;8(4):e70171. doi: 10.1002/cnr2.70171 (PMC11965703; doi:10.1002/cnr2.70171)
Supplement: Supplementary file 2 — Data S2. Supporting Information. [file CNR2-8-e70171-s001.docx]

**Supplementary Material**

eMethods. Search strategy for databases.

Figure S1. PRISMA flowchart outlining evidence search

Figure S2. Domain-wise Risk of bias assessment of included trials

Figure S3. Forest Plot showing Progression-Free Survival in the Overall Patient Population

Figure S4. Forest Plot showing Overall Survival in the Overall Patient Population

Figure S5. Forest Plot showing OR for MRD negativity rate in the Overall Patient Population

Figure S6. Forest Plot showing OR for ORR in the Overall Patient Population

Figure S7. Forest Plot showing OR for sCR in the Overall Patient Population

Figure S8. Forest Plot showing OR for CR or better in the Overall Patient Population

Figure S9. Forest Plot showing OR for VGRR or better in the Overall Patient Population

Figure S10. Forest Plot showing OR for Grade 3 or higher adverse events in the Overall Patient Population

Figure S11. Forest Plot showing OR for neutropenia in the Overall Patient Population

Figure S12. Forest Plot showing OR for infections in the Overall Patient Population

Figure S13. Forest Plot showing OR for thrombocytopenia in the Overall Patient Population

Figure S14. Forest Plot showing OR for lymphopenia in the Overall Patient Population

Figure S15. Forest Plot showing OR for anemia in the Overall Patient Population

Figure S16. Subgroup analysis of Progression-Free Survival by cytogenetic risk group

Figure S17. Subgroup analysis of MRD negativity rate by cytogenetic risk group

Figure S18: Forest Plot showing subgroup analysis for MRD negativity rate by number of cytogenetic high-risk features

Figure S19. Forest Plot showing subgroup analysis for MRD negativity rate by Gender.

Figure S20.Forest Plot showing subgroup analysis for MRD negativity rate Type of MM

Figure S21.Forest Plot showing subgroup analysis for MRD negativity rate by ECOG Score.

Figure S22.Forest Plot showing subgroup analysis for MRD negativity rate by ISS staging (1 vs 2).

Figure S23.Forest Plot showing subgroup analysis for MRD negativity rate by ISS staging (1 vs 3)

Figure S24.Forest Plot showing subgroup analysis for MRD negativity rate by ISS staging (2 vs 3)

Table S1. Outcome matrix for subgroups assessed in the included trials.

Table S2. Outcome definition in the included trials

Table S3. Definition of high-risk population in the included trials

**eMethods: Search strategy for databases.**

| **Database** | **Search Strategy** | **Articles** |
| --- | --- | --- |
| **Medline** | ((“multiple myeloma” or “myeloma” or “plasma cell neoplasm” OR “plasma cell myeloma” OR “multiple myelomas” OR “plasma cell dyscrasia” OR “plasma cell myeloma”) AND (“daratumumab” OR “isatuximab” OR “anti-CD38 antibody” OR “monoclonal antibody” OR “bortezomib” OR “proteasome inhibitor” OR “immunomodulatory drug” OR “lenalidomide” OR “dexamethasone” OR “prednisone” OR “betamethasone” OR “methylprednisolone” OR “velcade” OR “thalidomide” OR “carfilzomib”)) | 7,160 |
| **SCOPUS** | ( TITLE-ABS-KEY ( “multiple myeloma” OR “myeloma” OR “plasma cell neoplasm” OR “plasma cell myeloma” OR “multiple myelomas” OR “plasma cell dyscrasia” OR “plasma cell myeloma” OR “plasma cell dyscrasias” ) AND TITLE-ABS-KEY ( “daratumumab” OR “isatuximab” OR “anti-CD38 antibody” OR “monoclonal antibody” OR “bortezomib” OR “proteasome inhibitor” OR “immunomodulatory drug” OR “lenalidomide” OR “dexamethasone” OR “prednisone” OR “betamethasone” OR “methylprednisolone” OR “velcade” OR “thalidomide” OR “carfilzomib” ) AND ( LIMIT-TO ( LANGUAGE , “English” ) ) ) | 3,610 |
| **EMBASE** | (("multiple myeloma" or "myeloma" or "plasma cell neoplasm" or "plasma cell myeloma" or "plasma cell dyscrasia" or "plasma cell myeloma" or "plasma cell dyscrasias") and ("daratumumab" or "isatuximab" or "anti-CD38 antibody" or "monoclonal antibody" or "bortezomib" or "proteasome inhibitor" or "immunomodulatory drug" or "lenalidomide" or "dexamethasone" or "prednisone" or "betamethasone" or "methylprednisolone" or "velcade" or "thalidomide" or "carfilzomib")) | 992 |

**Figure S1: Preferred Reporting Items for Systematic Reviews and Meta-Analysis (PRISMA) flowchart outlining evidence search.**

Records identified through electronic database

(n =11,762)

Records identified through manual search (n=1)

**Identification**

Duplicate records removed

(n = 1,641)

Records after de-duplication

(n = 10,122)

**Screening**

**Screening**

Titles and abstracts screened

(n = 10,122)

Excluded by titles and abstracts

(n =10,104)

Full texts assessed for eligibility

(n =18)

Excluded by full-text review

(n= 11)

Studies included in systematic review

(Trials = 5; References=7)

**Included**

Studies included in meta-analysis

(Trials = 5; References=7)

Figure S2. Domain-wise Risk of Bias assessment for included trials.

Figure S3. Forest Plot showing Progression-Free Survival in Overall Patient Population


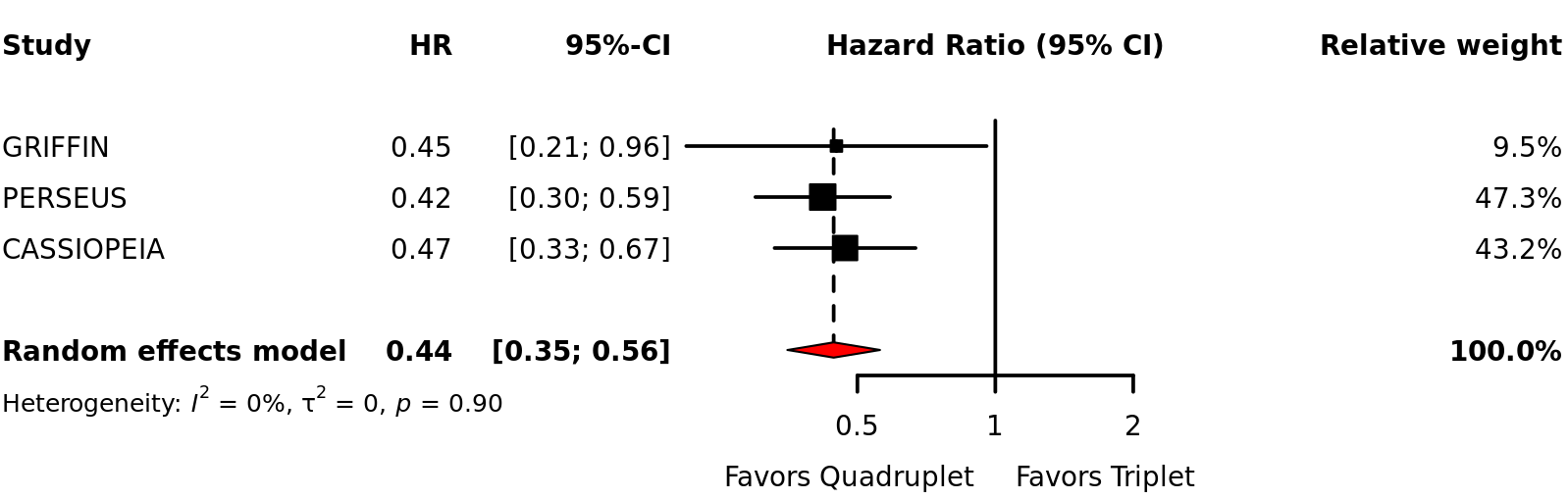


Figure S4. Forest Plot showing Overall Survival in Overall Patient Population


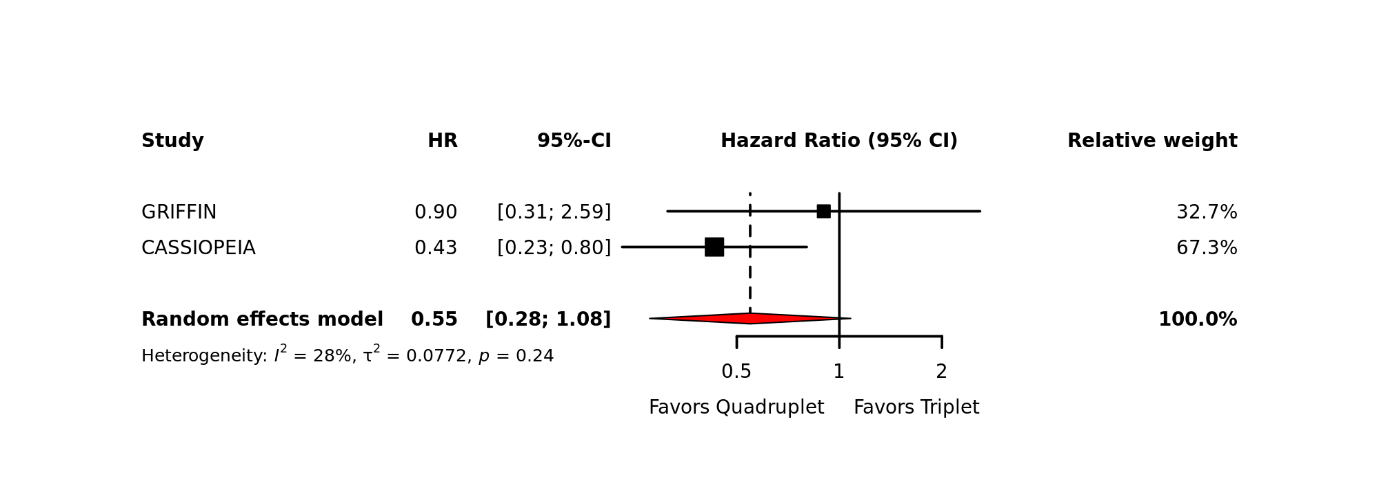


Figure S5. Forest Plot showing OR for MRD negativity rate in the Overall Patient Population


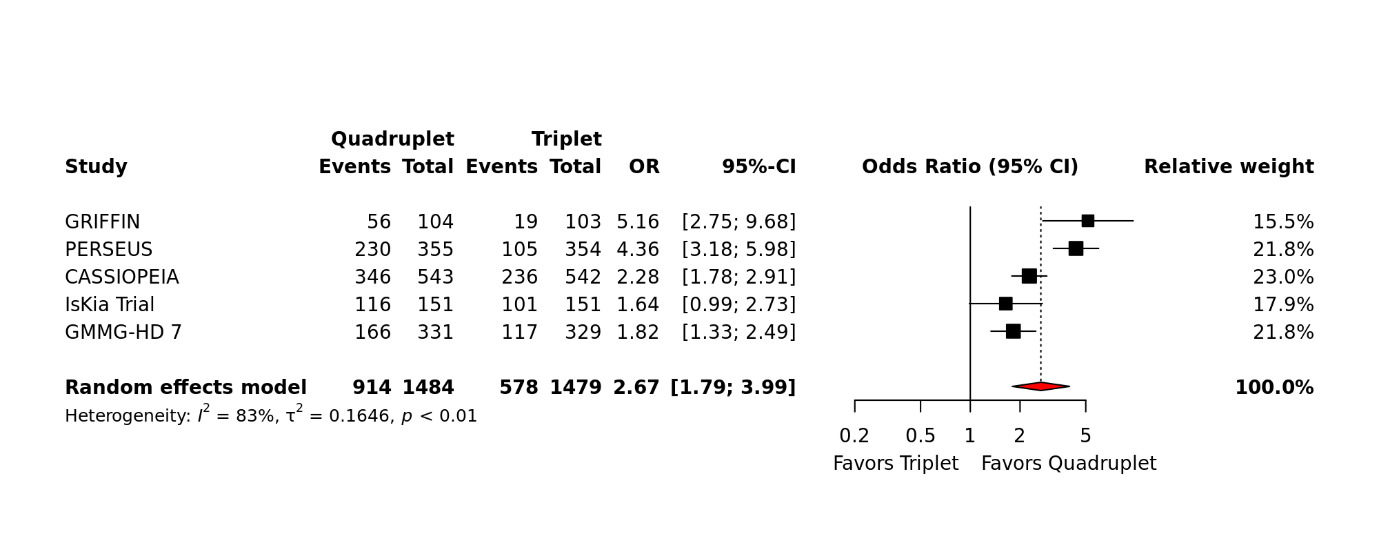


Figure S6. Forest Plot showing ORR in the Overall Patient Population


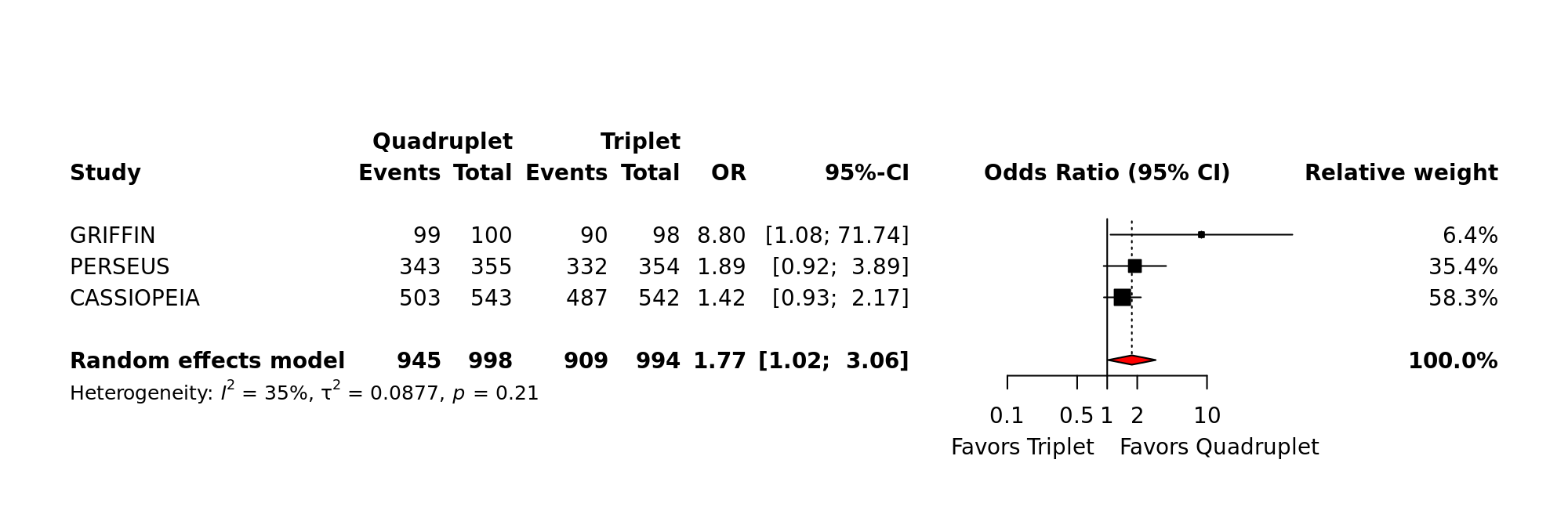


Figure S7. Forest Plot showing OR for sCR in the Overall Patient Population.


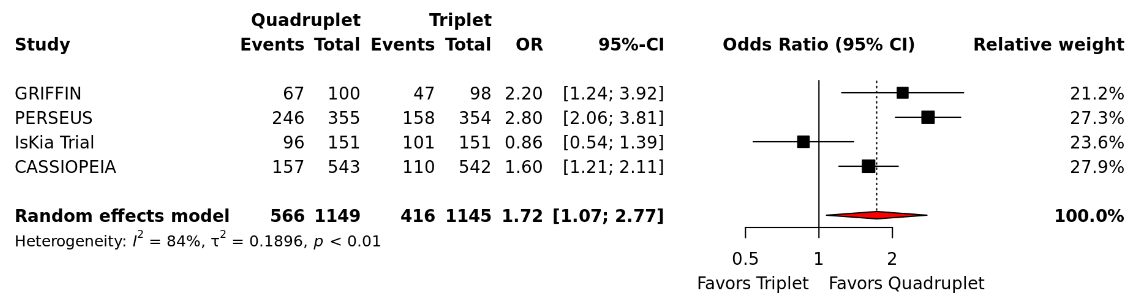


Figure S8. Forest Plot showing OR for CR or better in the Overall Patient Population


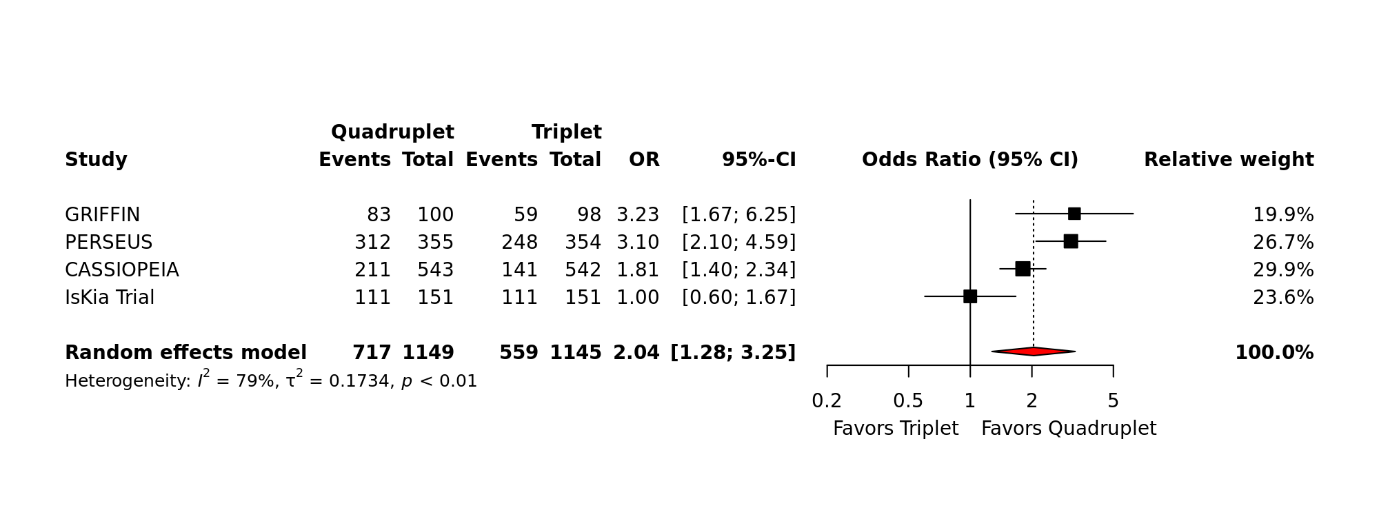


Figure S9. Forest Plot showing OR for VGRR or better in the Overall Patient Population


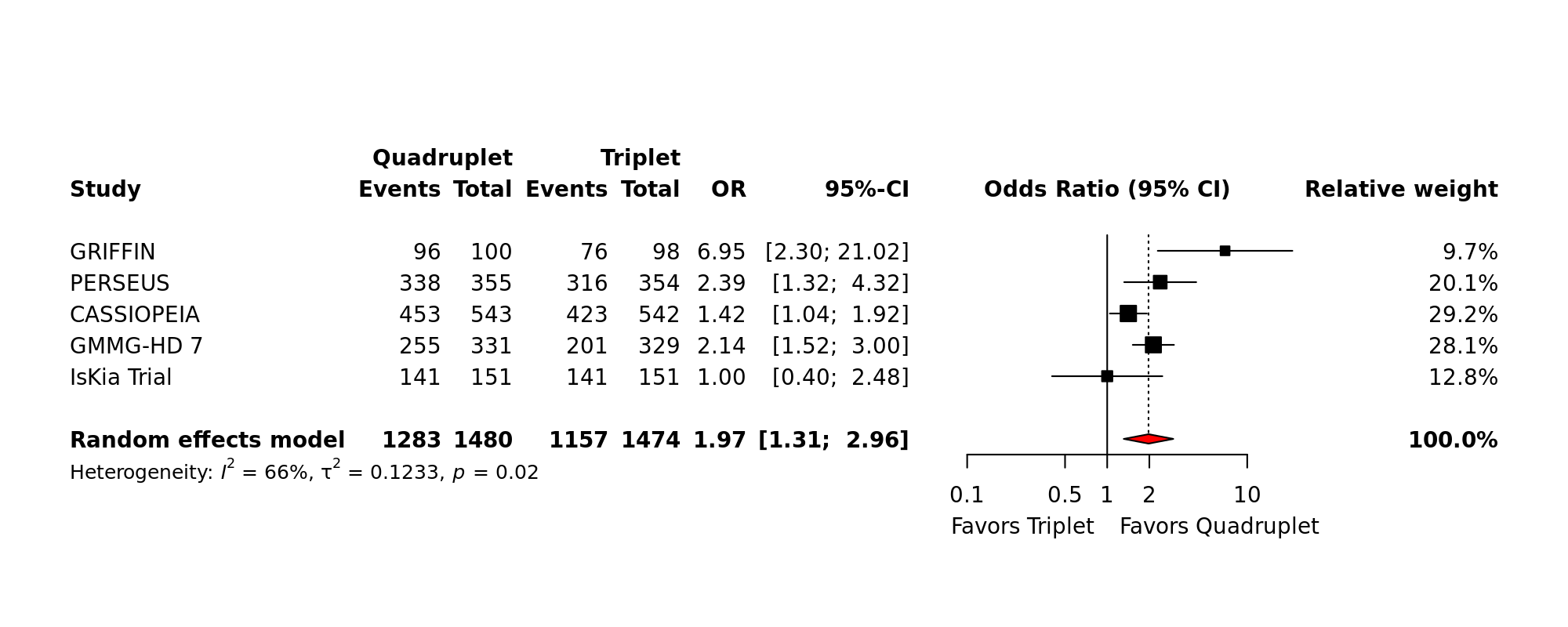


Figure S10. Forest Plot showing OR for Grade ≥ 3 adverse events in the Overall Patient Population.


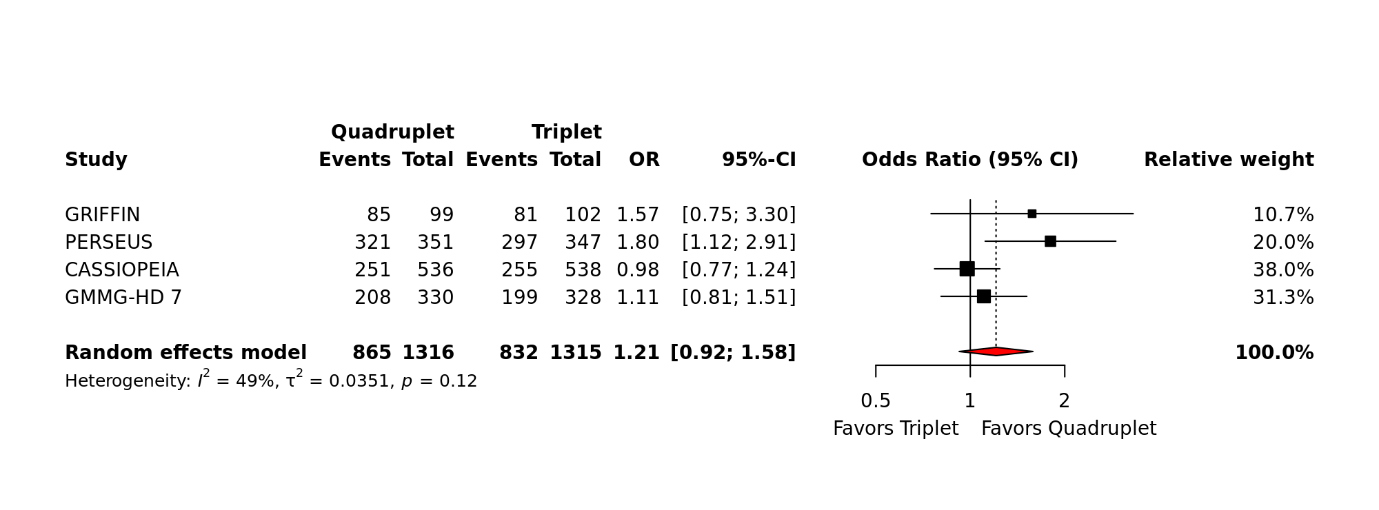


Figure S11. Forest Plot showing OR for Neutropenia in the Overall Patient Population


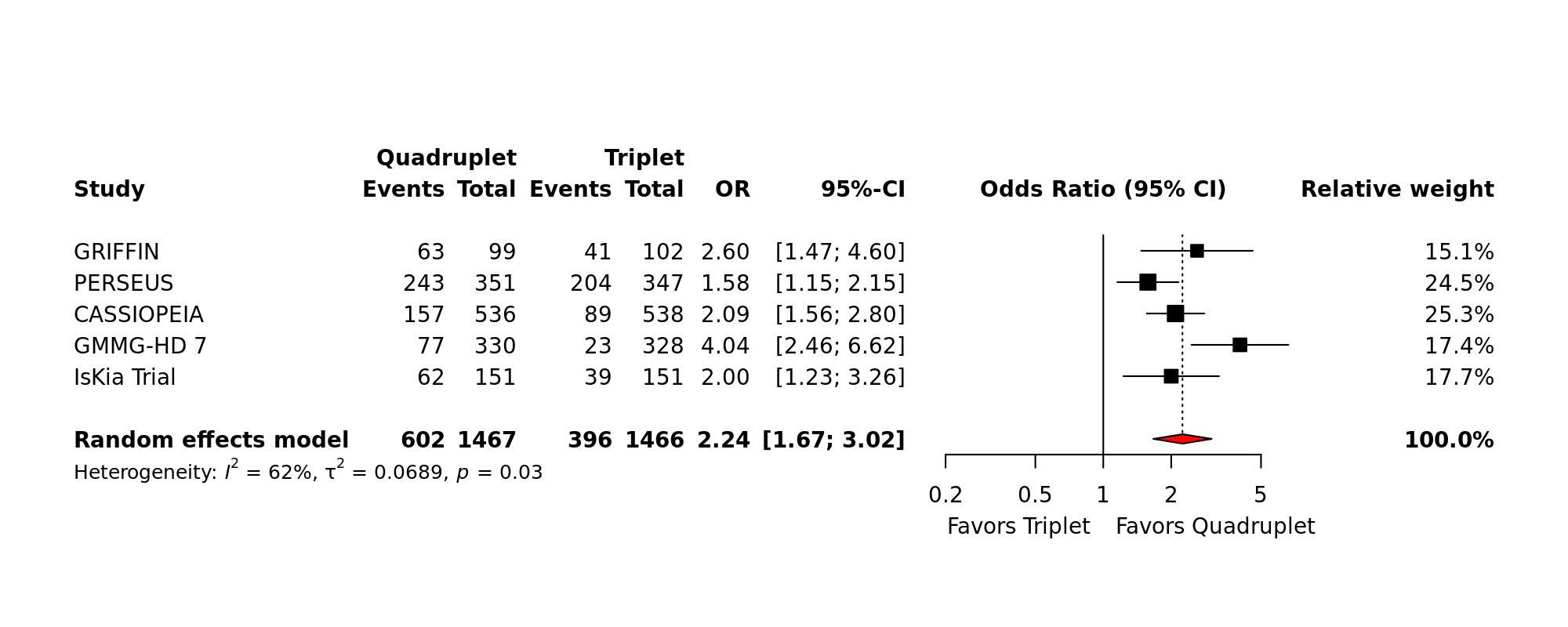


Figure S12. Forest Plot showing OR for infections in the Overall Patient Population.


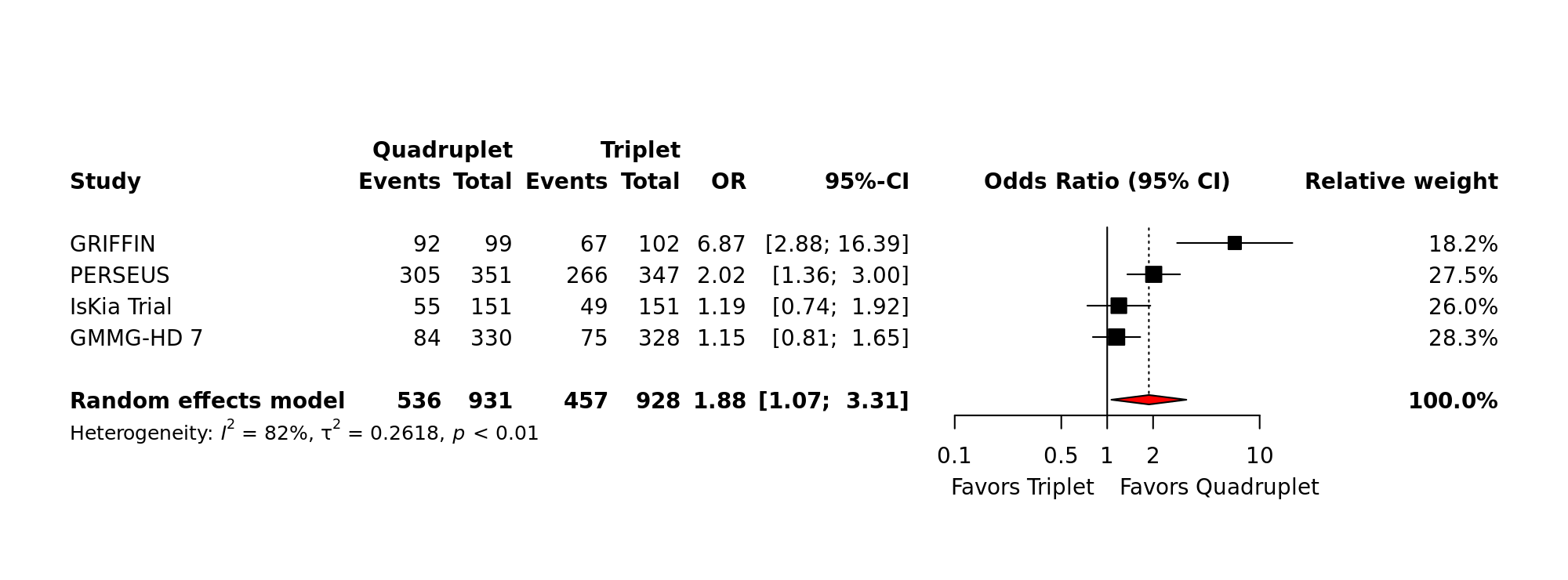


Figure S13. Forest Plot showing OR for thrombocytopenia in the Overall Patient Population.


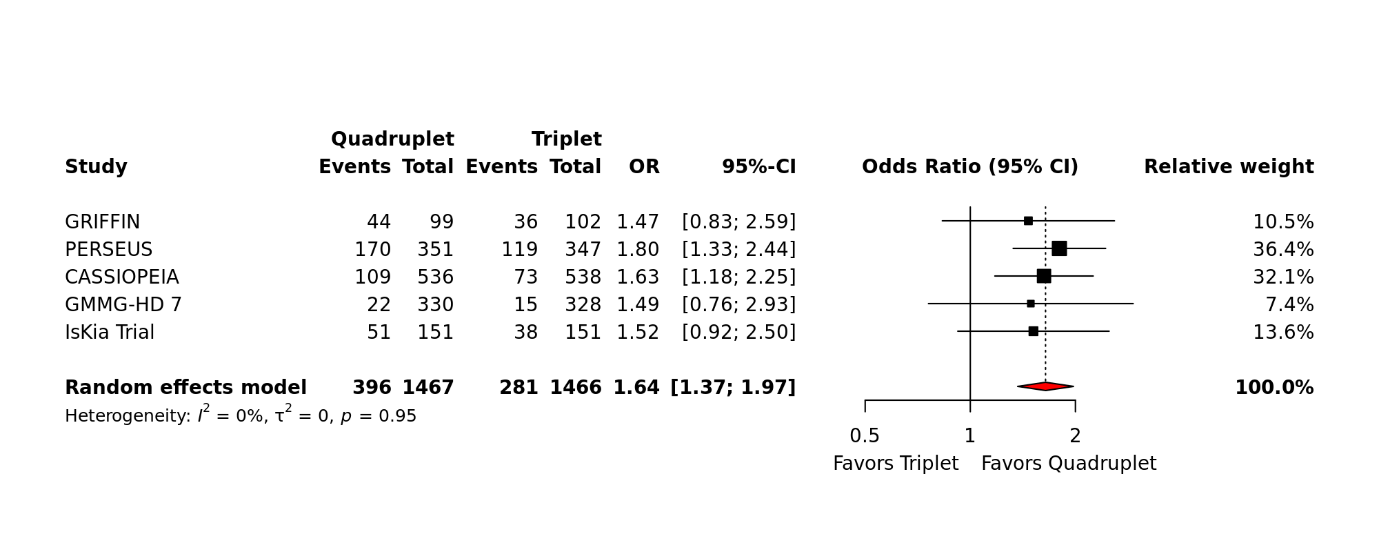


Figure S14. Forest Plot showing OR for lymphopenia in the Overall Patient Population.


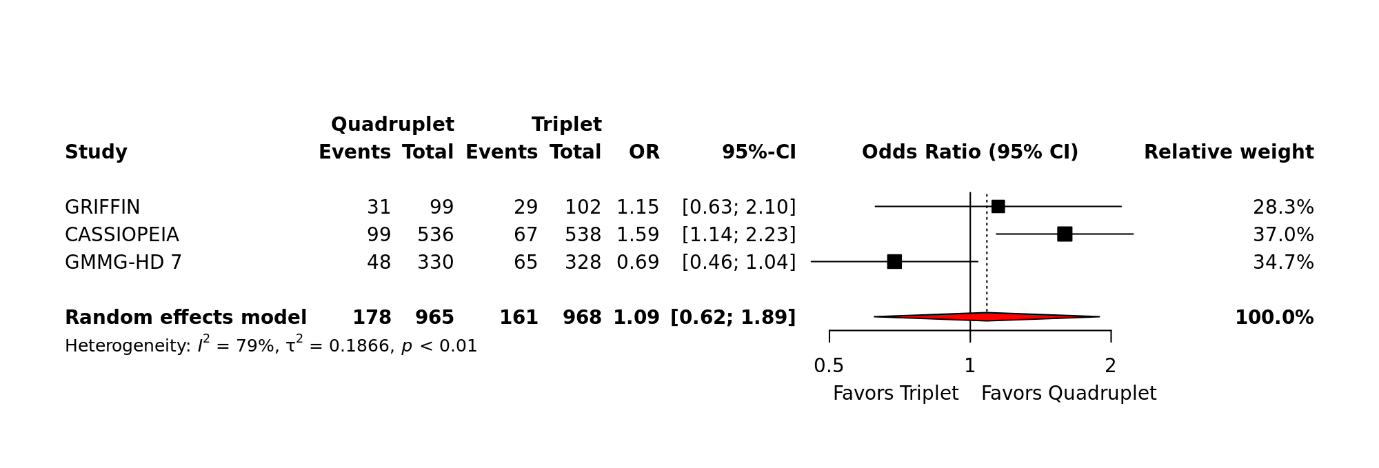


Figure S15. Forest Plot showing OR for anemia in the Overall Patient Population.


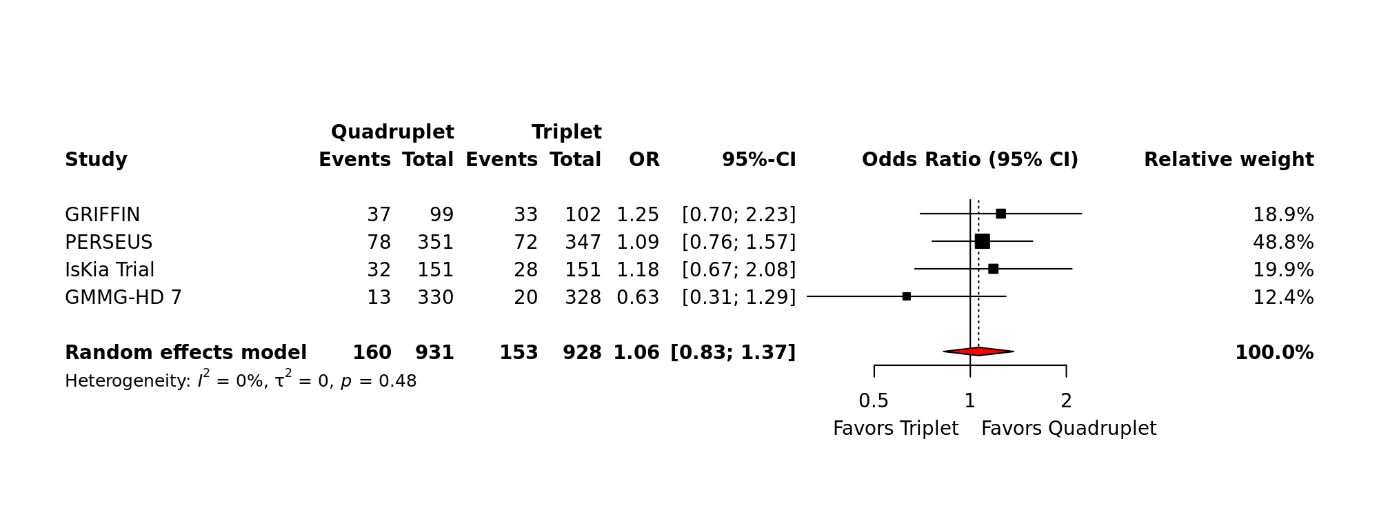


Figure S16. Forest plot showing PFS subgroup analysis by cytogenetic risk group.


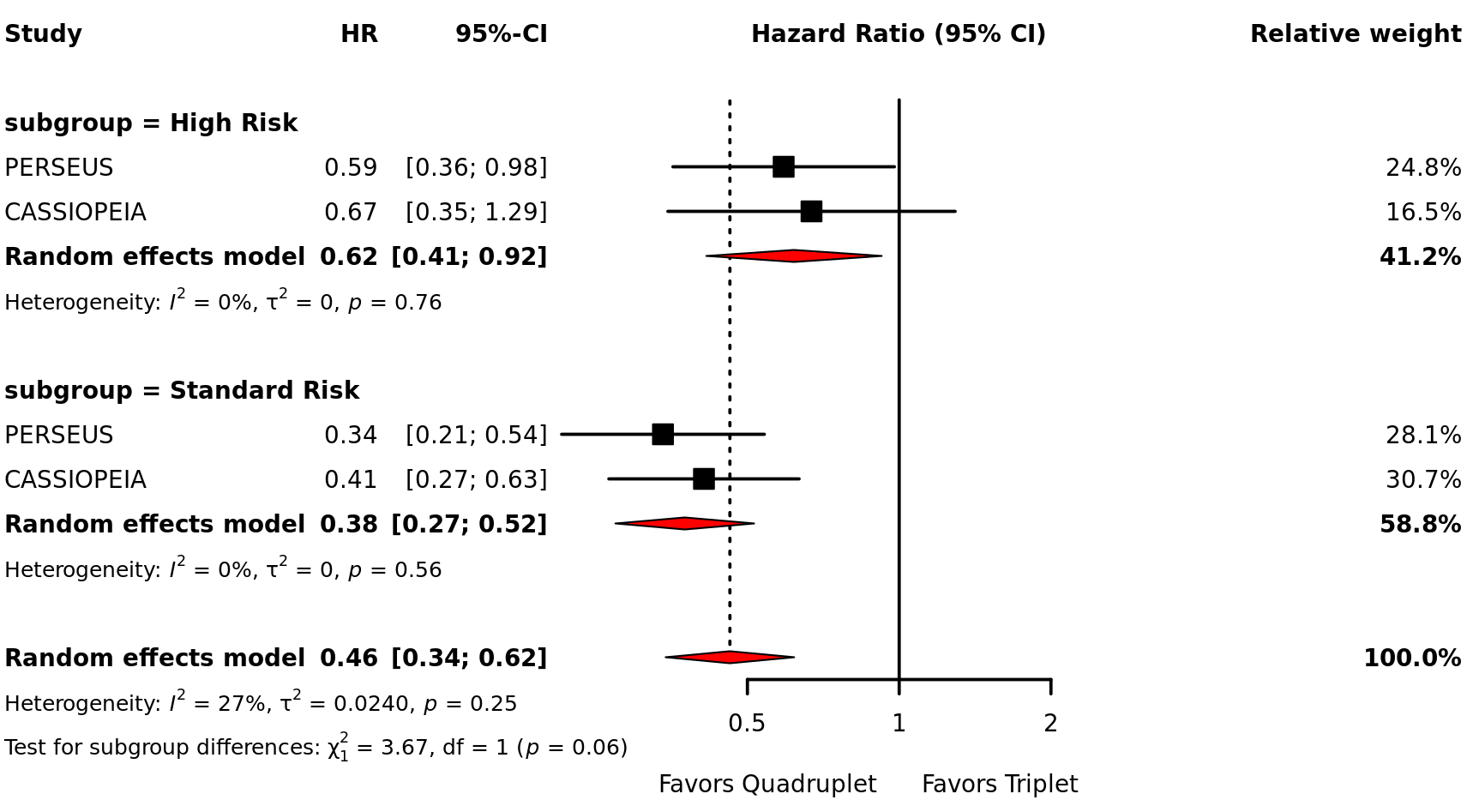


Figure S17. Forest Plot showing subgroup analysis for MRD negativity rate by cytogenetic risk group.


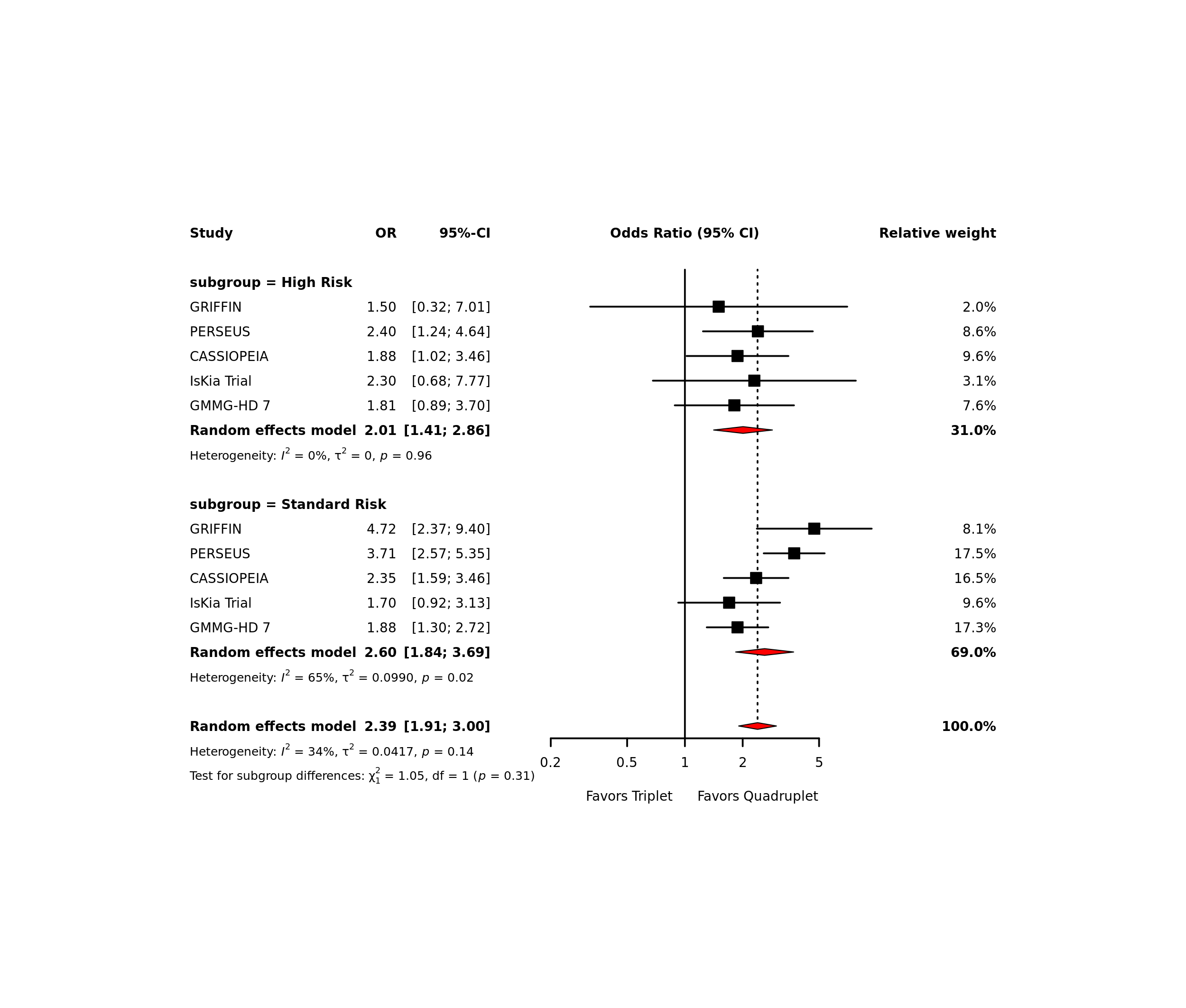


PERSEUS trial also reported patients with indeterminate risk. For the purpose of analysis, those patients

were included in the SR group.

Figure S18: Forest Plot showing subgroup analysis for MRD negativity rate by number of cytogenetic high-risk features


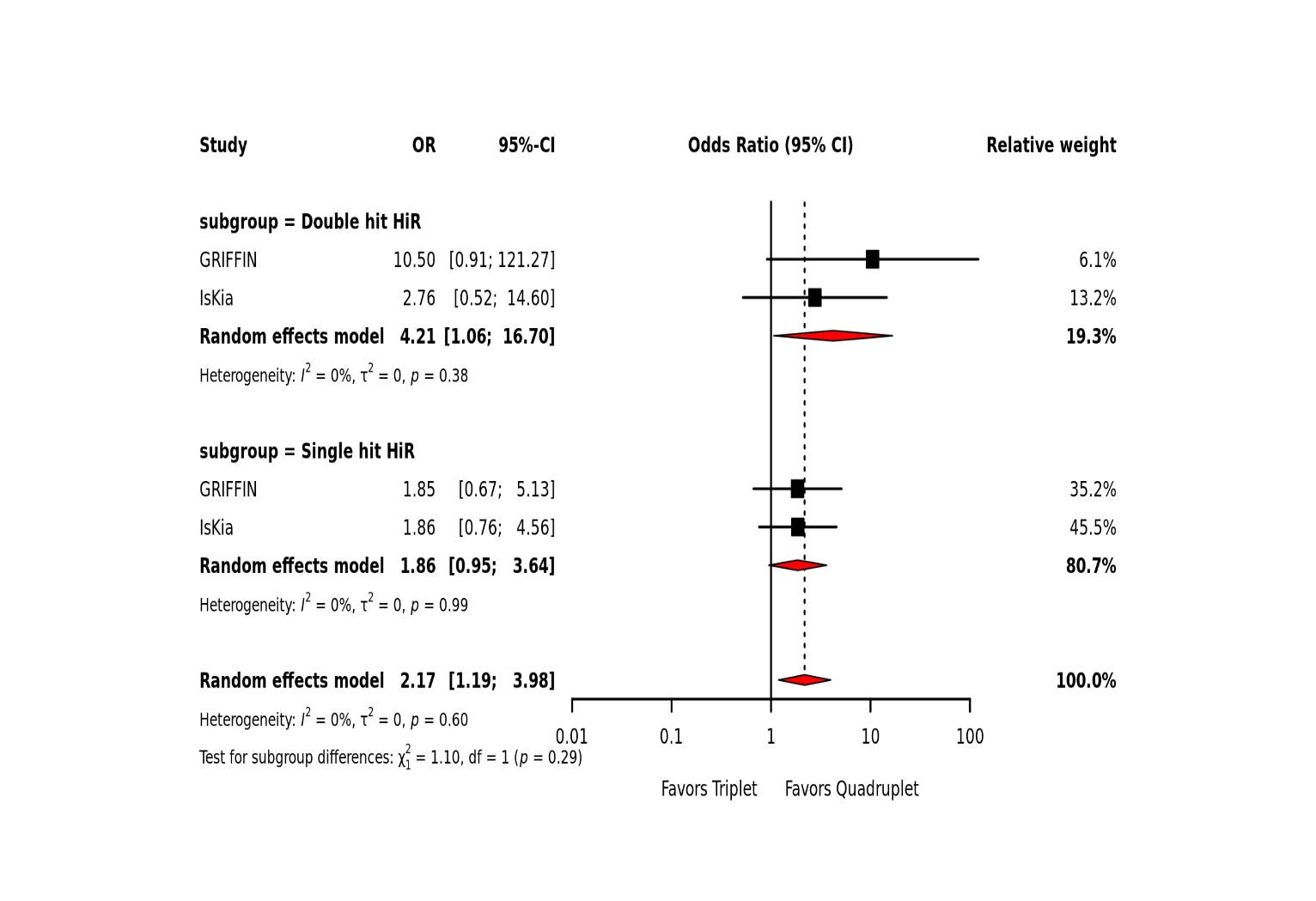


Figure S19: Forest Plot showing subgroup analysis for MRD negativity rate by Gender.


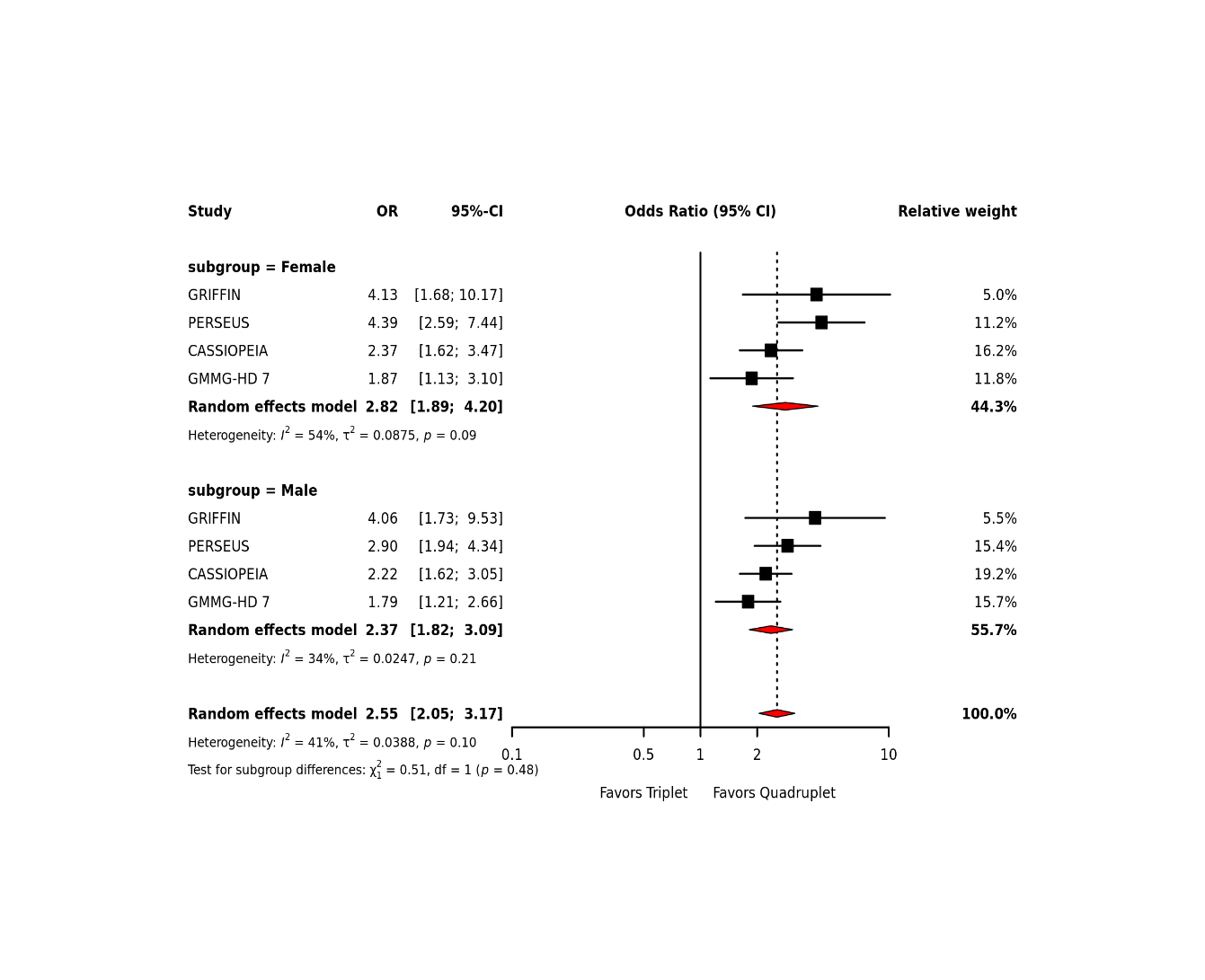


Figure S20.Forest Plot showing subgroup analysis for MRD negativity rate Type of MM


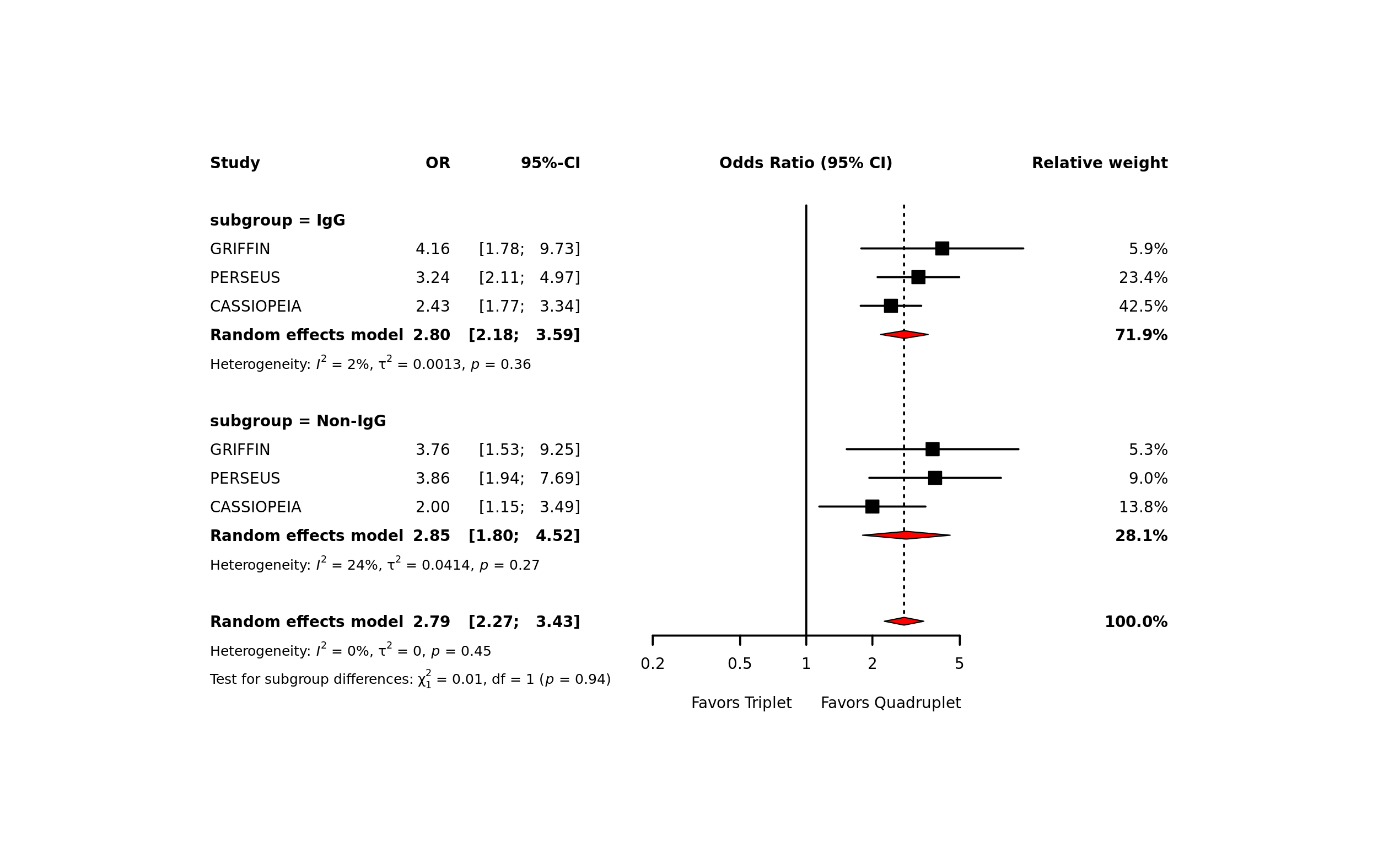


Figure S21.Forest Plot showing subgroup analysis for MRD negativity rate by ECOG Score.


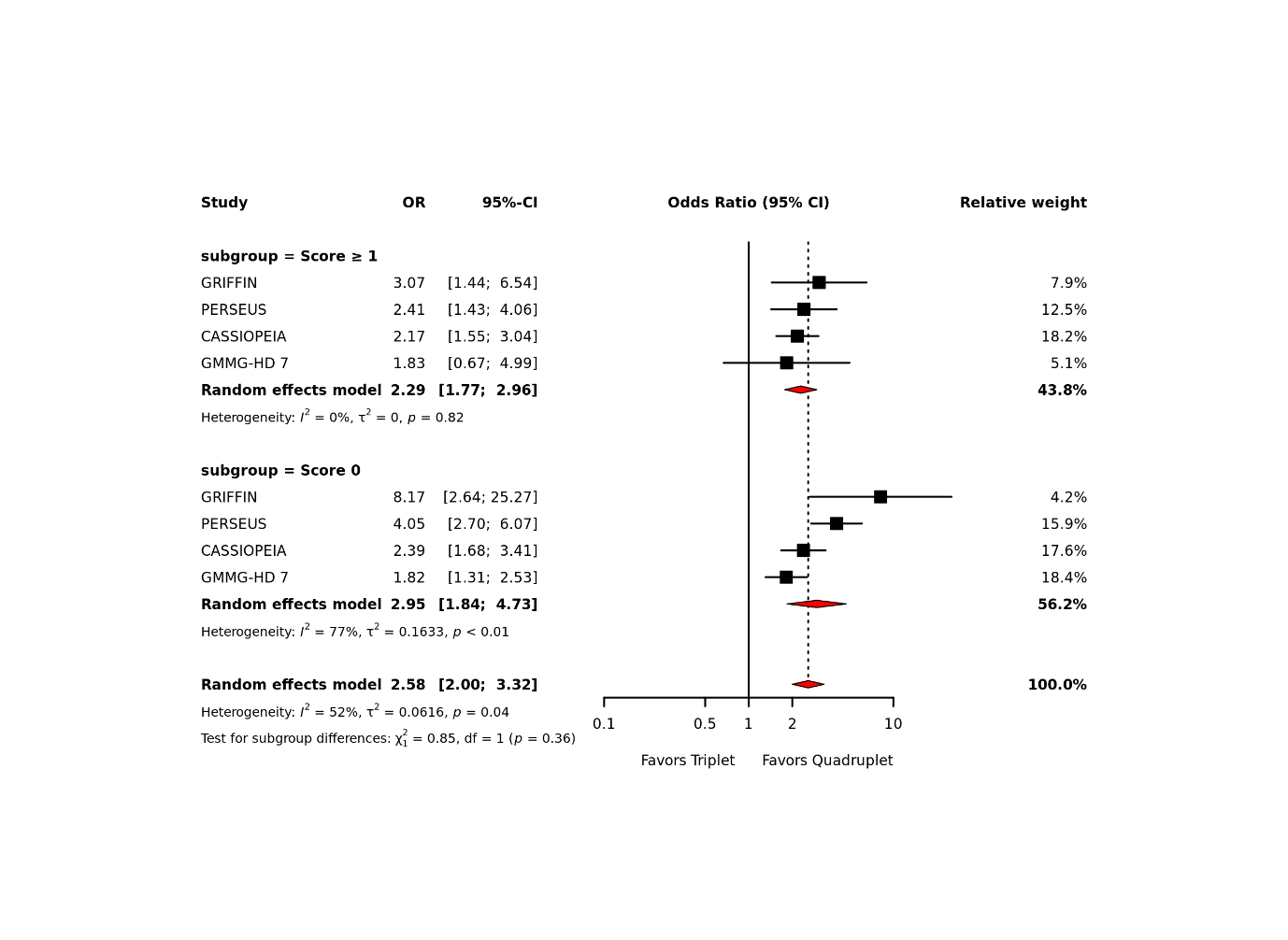


Figure S22.Forest Plot showing subgroup analysis for MRD negativity rate by ISS staging (1 vs 2).


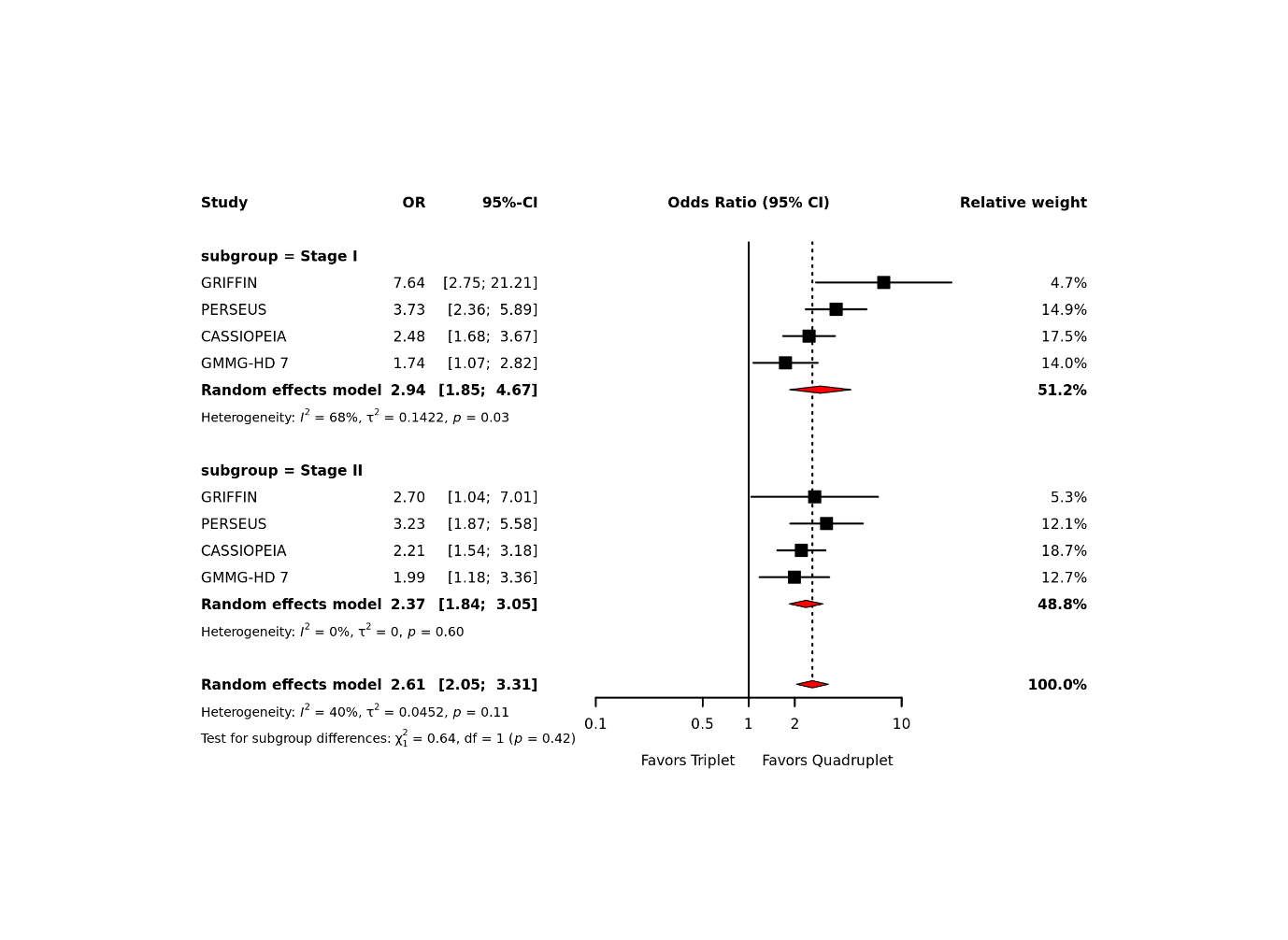


Figure S23.Forest Plot showing subgroup analysis for MRD negativity rate by ISS staging (1 vs 3)


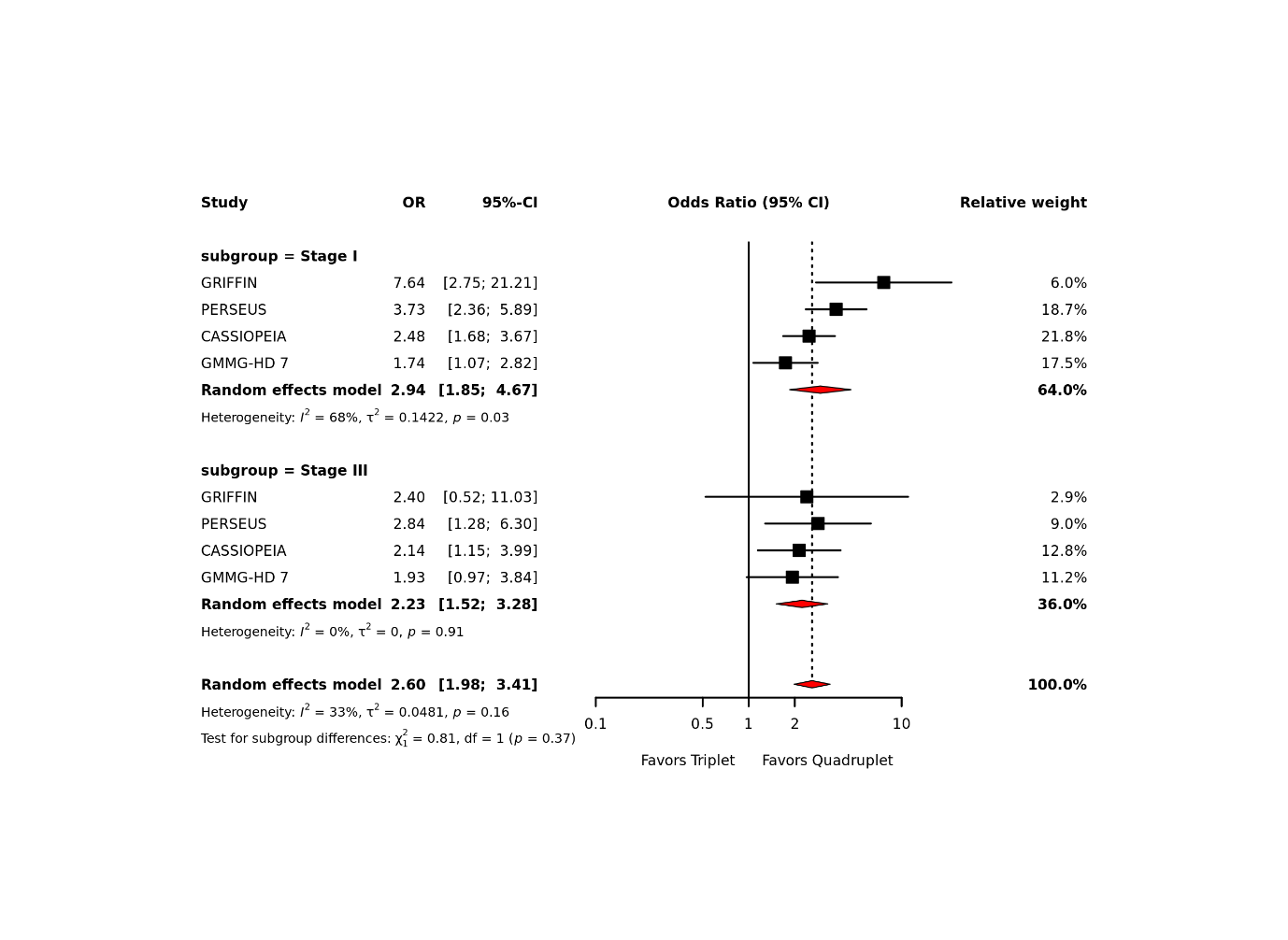


Figure S24.Forest Plot showing subgroup analysis for MRD negativity rate by ISS staging (2 vs 3).


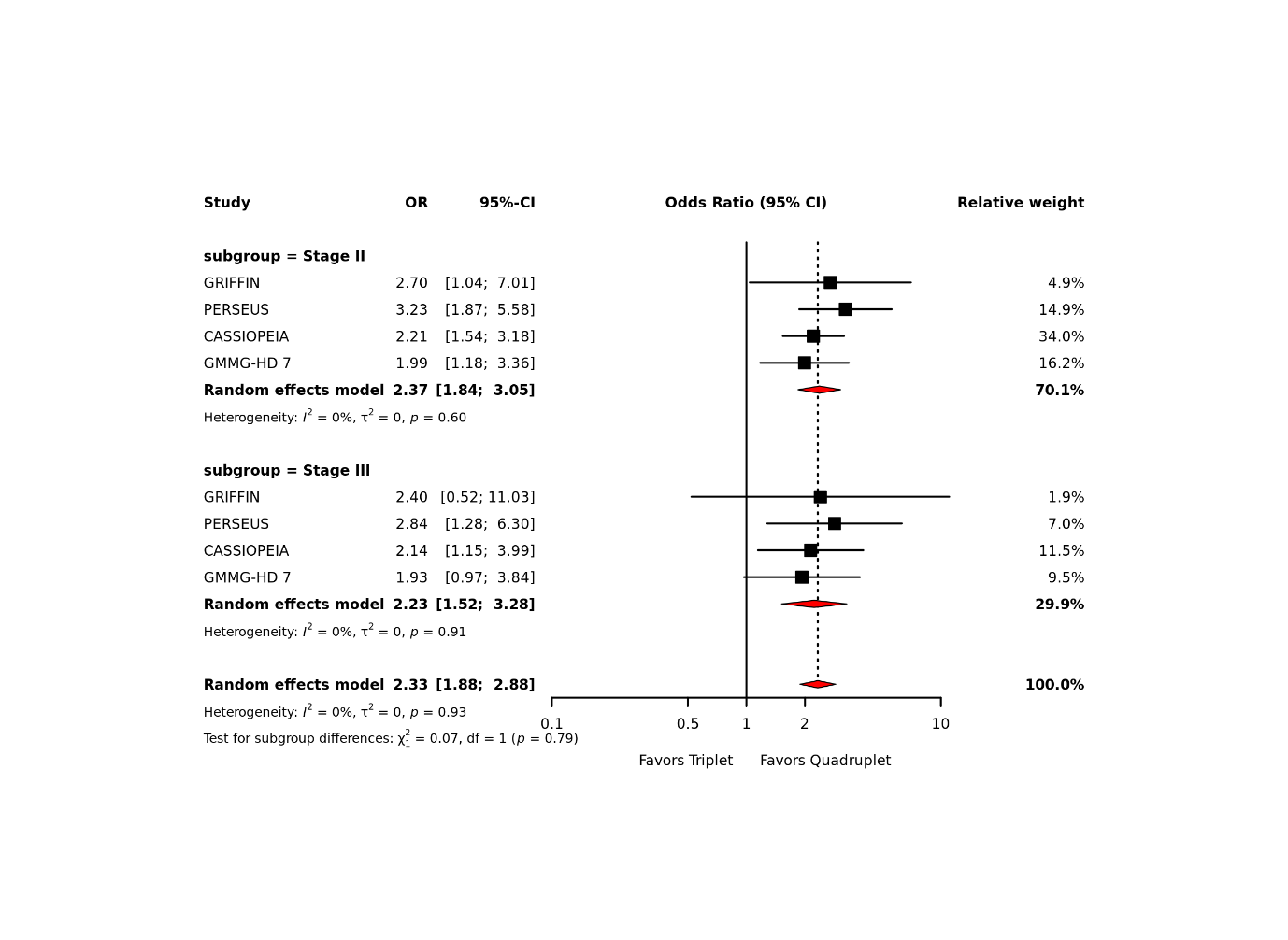


Table S1: Outcome matrix for subgroups assessed in the included trials.

Table S2: Outcome definition in the included trials.

| **GRIFFIN** | Objective response rate | The proportion of subjects who achieve partial response or better according to the International Myeloma Working Group criteria, by the respective time point. |
| --- | --- | --- |
|  | Stringent complete response rate | The proportion of subjects who have achieved sCR, according to the International Myeloma Working Group criteria. |
|  | VGPR or better rate | The proportion of subjects achieving VGPR or better, according to the International Myeloma Working Group criteria, by the respective time point. |
|  | Overall CR rate | The proportion of subjects who achieve CR (or sCR), according to the International Myeloma Working Group criteria, by the respective time point. |
|  | MRD negativity rate | The proportion of subjects who achieve MRD negative status by the respective time point by use of next-generation sequencing at a minimum sensitivity threshold of one in 100 000 cells [10^–⁵^]. |
|  | Progression-free survival | The duration from the date of randomization to the date of first documented evidence of progressive disease or death, whichever comes first. |
|  | Toxicity | Common Terminology Criteria for Adverse Events (CTCAE) version 4.03 was used for assessment. |
|  | Overall survival | The duration from the date of randomization to the date of the subject’s death. |
| **PERSEUS** | Overall complete response or better rate | The percentage of patients in the intention-to-treat population who achieved complete response or stringent complete response status at any time during the study per the International Myeloma Working Group criteria. |
|  | Very good partial response or better | The percentage of patients in the intention-to-treat population who achieved very good partial response or beteer status at any time during the study per the International Myeloma Working Group criteria. |
|  | Overall MRD-negativity rate | The proportion of patients in the intention-to-treat population who achieved both MRD negativity (at or below a sensitivity threshold of 10^–5^) by bone marrow aspirate and a complete response or better at any time after the date of randomization during the study. |
|  | Progression-free survival | The time from the date of randomization to the date of first disease progression according to the International Myeloma Working Group response criteria or death due to any cause, whichever occurred earlier. |
|  | Overall survival | The time from the date of randomization to the date of death due to any cause. |
|  | Toxicity | Common Terminology Criteria for Adverse Events (CTCAE) version 5 was used for assessment. |
| **CASSIOPEIA** | Stringent complete response rate | The percentage of patients in the intention-to-treat population achieving or maintaining a stringent complete response, as assessed by centralised analysis according to International Myeloma Working Group response criteria assessed at 100 days after ASCT or (or immediately after consolidation if >100 days) |
|  | Rate of complete response or better | The proportion of patients in the intention-to-treat population achieving or maintaining complete response or better, as assessed by centralised analysis in accordance with the International Myeloma Working Group criteria. |
|  | MRD negativity rate | The proportion of patients in the intention-to-treat population achieving minimal residual disease–negative status, in accordance with the International Myeloma Working Group criteria. |
|  | Progression-free survival | The time from the initial randomisation to either confirmed progressive disease, in accordance with the International Myeloma Working Group criteria, or death, whichever occurred first. |
|  | Toxicity | Common Terminology Criteria for Adverse Events (CTCAE) version 4 was used for assessment. |
|  | Progression free survival | The time from randomisation to progression or death from any cause, whichever occurred first. |
|  | Overall survival | The time from randomisation to time of death from any cause. |
|  | Toxicity | Common Terminology Criteria for Adverse Events (CTCAE) version 4 was used for assessment. |
| **GMMG-HD 7** | Stringent complete response | CR plus normal FLC ratio and absence of clonal cells in bone marrow by immunohistochemistry or immunofluorescence. |
|  | CR | Negative immunofixation on the serum and urine and disappearance of any soft tissue plasmacytomas and < 5% plasma cells in bone marrow |
|  | VGPR | Serum and urine M-protein detectable by immunofixation but not on electrophoresis or 90% or greater reduction in serum M-protein plus urine M-protein level < 100mg per 24h. When the only method to measure disease is by serum FLC levels, very good partial response requires a ≥90% decrease in the difference between involved and uninvolved FLC levels. |
|  | MRD negativity rate | Achievement of MRD negative status at a sensitivity cutoff of 1 tumour cell × 10⁵ nucleated cells, regardless of International Myeloma Working Group response status after the end of induction therapy. |
|  | Progression free survival | The time from first randomisation to progression or death from any cause, whichever occurred first. |
|  | Overall survival | The time from first randomisation to death from any cause |
|  | Complete response | Assessed according to International Myeloma Working Group criteria after induction therapy. |
|  | Toxicity | Common Terminology Criteria for Adverse Events (CTCAE) version 5 was used for assessment. |

Complete definitions were missing for Iskia trial due to limited data in the abstracts.

| **GRIFFIN** | The presence of del17p, t(4;14), or t(14;16), or a combination of these, among patients with available cytogenetic risk data, whereas revised high risk was defined as the presence of del17p, t(4;14), t(14;16), t(14;20), or gain/amp(1q21) (≥3 copies of chromosome 1q21) among those patients. |
| --- | --- |
| **PERSEUS** | The presence of del(17p), t (4;14), or t (14;16). |
| **CASSIOPEIA** | The presence of del17p or t [4;14]. |
| **GMMG-HD 7** | The presence of del (17) (p13), t (4;14) (p16; q32), or t(14;16) (q32;q23). |
| **IsKia Trial** | The presence of del(17p) and/or t (4;14) and/or t (14;16) |

Table S3: Definition of HiR MM in the included trials.
